# Bacterial immune priming and *Wolbachia*-mediated dengue blocking in *Aedes albopictus* mosquitoes

**DOI:** 10.1371/journal.pntd.0014807

**Published:** 2026-10-16

**Authors:** Abdullah A. Alomar, Dongmin Kim, Barry W. Alto, Eric P. Caragata

**Affiliations:** 1 Infectious Disease Vector Research Laboratory, Department of Plant Protection, College of Food and Agricultural Sciences, King Saud University, Riyadh, Saudi Arabia; 2 Florida Medical Entomology Laboratory, Department of Entomology & Nematology, Institute of Food and Agricultural Sciences, University of Florida, Vero Beach, Florida, United States of America; Instituto Leonidas e Maria Deane / Fundacao Oswaldo Cruz, BRAZIL

## Abstract

**Background:**

*Wolbachia* is a maternally-transmitted bacterial endosymbiont that is highly prevalent amongst arthropods. Many *Wolbachia* strains induce pathogen blocking, thereby protecting their hosts from pathogenic microbial infections. Blocking against pathogenic bacteria occurs in *Aedes aegypti* mosquitoes, where *Wolbachia* has been artificially established by microinjection. However, in *Drosophila* fruit flies, it occurs after oral bacterial challenge but not after systemic challenge via injection. Pathogen blocking has been linked to increased host immune activity associated with *Wolbachia* infection. This is similar in principle to immune priming, a memory immune response in insects, where sub-lethal exposure to a pathogen increases the ability to survive future infections. *Aedes albopictus* mosquitoes naturally harbor *Wolbachia*; however, the extent to which native *Wolbachia*-mediated pathogen blocking and immune priming influence host response to infection remains unclear. Accordingly, we sought to characterize the response of *Wolbachia*-infected *Ae. albopictus* to bacterial challenge, and to determine whether the combination of these microbes could hinder a subsequent infection with dengue virus (DENV).

**Methodology/principal findings:**

We challenged adult female *Ae. albopictus* with and without their native *Wolbachia* infection with a sub-lethal dose of four mosquito-associated bacteria: *Micrococcus, Staphylococcus* (gram positive), *Brevundimonas*, *Enterobacter* (gram negative). Assessing post-challenge mortality, we observed that *Wolbachia* infection reduced cumulative mortality and delayed the onset of senescence amongst mosquitoes challenged with each bacterium. However, this protective effect was stronger against gram positive bacteria. Bacteria-primed *Ae. albopictus* were then orally challenged with DENV. We observed that mosquitoes that experienced a pre-challenge with gram negative bacteria were associated with reduced DENV prevalence and viral load, whereas *Wolbachia*-infected mosquitoes exhibited further reductions in viral load.

**Conclusions/significance:**

We highlight novel characteristics of native *Wolbachia* infection in *Ae. albopictus* mosquitoes, with protection against bacterial-induced mortality, and interactions between pathogen blocking and bacterial priming that may depend on bacterial gram status. These findings provide insight into the immune interactions associated with native *Wolbachia* and build on our understanding of adaptive immunity in mosquitoes.

## Author summary

The immune response of insects is heavily linked to its microbiological history. Infections early in life and lifelong infections with beneficial microorganisms can both help an insect to more effectively recover from future infections. *Wolbachia pipientis* is a bacterium commonly found in insects that can protect against harmful bacteria, fungi, and viruses. In this study, we investigated the impact of co-infections with *Wolbachia*, mosquito-associated bacteria, and dengue virus, in *Ae. albopictus*, a mosquito species important to public health. We examined how the presence of *Wolbachia* helps to limit pathogen-induced death of mosquitoes after exposure to harmful bacteria. Also, a previous sublethal infection with bacteria in adult mosquitoes mitigates dengue virus infection following oral challenge. These beneficial immune responses are even more strongly exhibited among mosquitoes with a native *Wolbachia* infection. Our results demonstrate the versatility of microbial interactions in shaping immune responses in mosquitoes.

## Introduction

The gram negative bacterial endosymbiont *Wolbachia pipientis* is one of the most ubiquitous microorganisms in arthropods [1]. Its ability to manipulate host reproductive biology can facilitate the rapid spread of the symbiont in host populations, or bias sex ratios against males to promote maternal transmission [2]. This reproductive parasitism acts as a natural form of gene drive that allows *Wolbachia* to persist in insect populations even in the face of fitness costs incurred by the host [3,4]. *Wolbachia* can also positively impact host biology. For instance, enhanced reproductive capacity in *Trichogramma bourarachae* [5], improved juvenile performance in *Hylyphantes graminicola* [6], and metabolic provisioning in *Drosophila melanogaster* [7] and *Cimex lectularius* [8].

Many *Wolbachia* strains help their host to resist pathogen infections, an effect known as *Wolbachia*-mediated pathogen blocking. This encompasses multiple phenotypes including reduced or delayed mortality after exposure to a pathogenic microorganism [9,10], reduced prevalence of pathogen infection [11], reduced pathogen load [9,10], and reduced rates of pathogen transmission [12,13]. The phenotype was first observed in *D. melanogaster* fruit flies [9,10], but has also been characterized in mosquito species such as *Aedes albopictus* [14] and *Culex quinquefasciatus* [15,16], which harbor native *Wolbachia* infections.

Pathogen blocking has been most studied in *Aedes aegypti* mosquitoes, where *Wolbachia* transinfections have been artificially established through embryonic microinjection [17–19]. Across *Wolbachia*-infected *Ae. aegypti* lines, blocking has been observed against many arboviruses important to human health, including dengue and Zika [11,12,18,20,21], and parasites of veterinary importance like the avian malaria *Plasmodium gallinaceum* [11] and *Brugia pahangi* filarial nematodes [22]. Pathogen blocking has proven to be a critical element of population replacement interventions that have spread *Wolbachia* into *Ae. aegypti* populations [23,24], leading to associated reductions in dengue risk in treated areas [25,26].

*Wolbachia* can also protect their hosts against certain pathogenic bacteria and fungi. However, this effect is contingent on multiple factors, including the *Wolbachia* strain, the route of pathogen delivery, and infection, and whether the *Wolbachia* infection is native or artificial. Transinfections with the *w*MelPop strain in *Ae. aegypti* have consistently demonstrated pathogen blocking against diverse bacteria following intrathoracic inoculation [22,27]. In contrast, *w*Mel-infected *Ae. aegypti* exhibit bacterium-specific variation in blocking efficacy [27]. Studies in *D. melanogaster* and *Drosophila simulans* have generally reported an absence of protection against pathogenic bacteria delivered intrathoracically [28–30]. Nevertheless, blocking was seen in *D. melanogaster* after oral challenge with *Pseudomonas aeruginosa* [28], and after systemic challenge with various pathogenic fungi [31].

The pathogen blocking mechanism is uncertain, but is likely multifactorial, as well as host species-, *Wolbachia* strain-, and pathogen-specific [32–35]. In different host-symbiont combinations, blocking has been linked to increased Toll pathway immune effector gene activity [36,37], the RNA interference pathway [38], a dysregulated oxidative stress response [36], altered metabolism [39–41], degradation of viral RNA [42], and manipulation of host processes important to arboviral replication cycles [43–45].

*Wolbachia*-mediated immune activation of the Toll pathway is more characteristic of *Wolbachia* transinfections in mosquitoes than of native *Wolbachia* infections. It involves increased expression of antimicrobial peptide genes, particularly cecropins and defensins, as well as other bacterial binding proteins [36,37,46]. This effect has been linked to both *Wolbachia*-mediated induction of reactive oxygen species [36], and *Wolbachia*-mediated manipulation of cholesterol [47,48]. Immune activation is attenuated in mosquitoes with native *Wolbachia* infections [49], and there is no evidence it is involved in pathogen blocking in *Drosophila* [30,35]. It is theorized to contribute to pathogen blocking through constitutive activation of the immune system enabling more effective response to future infections. This parallels immune priming [50], an adaptive immunological response in insects where an initial challenge with a pathogen facilitates more effective clearance of subsequent infections. Immune priming can occur for combinations of bacteria, fungi, and viruses [51,52]. It can act transstadially [53] or trans-generationally [54], making it a powerful tool for insects to resist the detrimental effects of microbial pathogens. Mechanistically, it has been associated with both innate and cellular immune pathways [55–57]. In female *Armadillidium vulgare*, *Wolbachia* infection improved survival after *Salmonella enterica* challenge, and displayed an age-dependent increase in blocking strength if pre-challenged with the same bacterium [58]. This finding highlights the potential for additive interactions between immune priming and pathogen blocking.

In *Ae. albopictus*, arbovirus blocking associated with the native *w*AlbA and *w*AlbB *Wolbachia* infections is either weak [14], conditional [15], or absent [59]. Weaker pathogen blocking with native infections is theorized to be the natural consequence of a long-term association between *Wolbachia* and host tending more towards mutualism and tolerance, with an accompanying attenuation of costly immune effects [60,61]. It is currently unclear whether native *Wolbachia* infections can protect their host against pathogenic bacterial infection. Likewise, it is unclear whether an initial microbial challenge in a mosquito harboring *Wolbachia* can result in stronger pathogen blocking during a second challenge. Here, we examine microbe-microbe effects in *Ae. albopictus*, assessing the role of *Wolbachia* in the response to bacterial infection, and to dengue virus infection after pre-challenge with sub-lethal bacterial infections.

## Materials and methods

### Mosquito rearing

The *Aedes albopictus* mosquitoes used in this experiment were originally collected in Vero Beach, Florida during 2021. Since collection, they have been maintained under standard insectary conditions in a climate-controlled chamber (26 ± 2°C, RH – 60–80%, photoperiod - 12h light: 12h dark). Colony larvae held in rearing trays containing distilled water were fed an equal mixture of dried *Saccharomyces cerevisiae* yeast and lactalbumin (150 mg) every four days until pupation. Pupae were collected daily from each tray and transferred to plastic cups containing distilled water and placed inside cages (Volume = 0.027m^3^). Newly emerged adults were provided with 10% sucrose solution.

### Wolbachia infection

Experiments in this study utilized two *Ae. albopictus* colonies: a wildtype colony naturally infected with *Wolbachia* (WT), and a second line derived from the WT colony where the *Wolbachia* infection was cleared by treatment with the antibiotic tetracycline (Tet). To generate the Tet colony, adult WT mosquitoes were fed on 10% sucrose solution containing 1 mg/mL of tetracycline hydrochloride (Catalog No. AAB2140814, ThermoFisher Scientific, Waltham, MA, USA) for three consecutive generations. Following treatment, the *Wolbachia* infection status of both colonies was confirmed via quantitative PCR. For this assessment, DNA was extracted from 20 adult female mosquitoes per colony using the DNeasy Blood & Tissue Kit (Qiagen, Germantown, MD, USA), according to the manufacturer’s instructions.

*Wolbachia* screening was performed using 2x SsoAdvanced universal SYBR green supermix (Bio-Rad, Hercules, CA, USA) and measured on a CFX-96 real-time PCR detection system (Bio-Rad) using the following run profile: 95°C for 5 min, then 35 cycles of 95°C for 5 sec and 60°C for 30 sec, followed by a melt-curve analysis (65–95°C with 0.5°C increments, 2–5 sec/step). Reactions were performed in duplicate for each sample and contained 0.5 µl of each primer (2.5μM), 5µL of SYBR green, 2µL of extracted genomic DNA, and 2µL of nuclease-free water. Samples were screened using primers for the *Wolbachia* 16S ribosomal RNA gene (16S rRNA-F: GAGTGAAGAAGGCCTTTGGG; 16S rRNA-R: CACGGAGTTAGCCAGGACTTC [33], which confirmed complete absence of infection in the Tet line, and 100% prevalence of infection in the WT line. Additionally, all specimens from the WT colony were confirmed to harbor both the *w*AlbA and *w*AlbB *Wolbachia* strains via qPCR screening with strain-specific primers (*w*AlbA - cwAlbA_F: GTAGTATTTACCCCAGCAG, cwAlbA_R: ATCTGCACCAGTAGTTTCG; *w*AlbB - cwAlbB_F GCAATACCTATGCCGTTTA, cwAlbB_R: GACGAAGGGGATAGGTTAATATC) [62]. All primers were synthesized by Integrated DNA Technologies (Coralville, IA, USA).

To normalize the microbiota between the two colonies, samples of water were collected from WT larval rearing trays after the completion of pupation and used to inoculate Tet larval rearing trays containing second-instar larvae. Briefly, an aliquot of water was stored at 4°C in a 50mL conical tube. Prior to use, this solution was agitated by hand to homogenize any detritus, then 1mL aliquots were added to Tet larval trays on the second day post-hatch. This procedure was repeated for two consecutive generations prior to the initiation of experiments. Experiments were performed 4–5 generations after the completion of tetracycline treatment, which corresponded to 9–10 generations after colonization for the WT line.

### Pathogenic bacterial isolates

Bacterial strains used in this study were originally isolated from mosquito specimens collected in the Oslo Riverfront Conservation Area, Vero Beach, FL, USA during 2021. Mosquitoes were collected via CDC miniature light traps baited with dry ice. Mosquito specimens were identified to species using a morphological key [63]. Mosquito specimens were surface sterilized via submersion in 70% ethanol for 1 min, followed by two successive 1 min washes in molecular-grade water [64]. Individual mosquito specimens were then homogenized in sterile 1X PBS using a TissueLyser (Qiagen) and a sterile 2 mm glass bead. Each homogenate was diluted 1:10 in sterile 1X PBS, and spread onto LB agar plates via agitation with 5–10 sterile 5mm beads. After those beads were discarded, plates were maintained at 30°C in an incubator (CCC 2.5d, Santa Cruz Biotechnology, Dallas, TX, USA) for 48h. After this time, colonies with unique morphologies were isolated using a sterile loop and used to inoculate fresh LB agar plates, to generate monocultures.

Colonies from monoculture plates were used to inoculate tubes containing LB broth, which were grown for 16h at 30°C and 200 rpm on an incubated shaker (Benchmark). Bacterial stocks comprised of a 1:1 mixture of those cultures and 50% glycerol solution (Catalog No. NC0352573, Fisher Scientific) were maintained at -80°C. These stocks were used to inoculate further LB agar plates, which were shipped to GENEWIZ (South Plainfield, NJ, USA) for targeted 16s rRNA-based sanger sequencing of bacterial colonies via their in-house pipeline. The resulting sequences were compared against the GenBank database using the nucleotide BLAST function. We identified four bacterial isolates from distinct bacterial classes (two gram positive, and two gram negative) for use in this study: *Enterobacter* sp. (-) and *Brevundimonas* sp. (-), isolated from *Aedes taeniorhynchus* specimens, *Micrococcus luteus* (+), isolated from a *Culex nigripalpus* specimen, and *Staphylococcus hominis* (+), isolated from an *Anopheles crucians* mosquito. Sequences are provided in S1 File.

To assess the pathogenicity of these four isolates to *Ae. albopictus*, we challenged adult female WT mosquitoes (17–27 mosquitoes per treatment, 2 replicate experiments, for a total of 608 specimens) aged at five days-post eclosion with either sterile 1X PBS (control) or one of three different concentrations of washed bacterial cells via intrathoracic injection using a Nanoject III handheld injector system (Drummond Scientific, Broomall, PA, USA). Systemic microbial injections in insects commonly occur via the cuticle or via wounding and are often examined in experimental settings via stabbing or injection [65–68]. Briefly, after 16h of growth in LB broth (30°C, 200rpm), culture OD_600_ values were measured using a spectrophotometer (Genesys 10S, Thermo Fisher Scientific), cells were then centrifuged (6000rpm, 4°C) and washed three times in 1X sterile PBS. On the final wash, cells were resuspended to (A) a volume equivalent to OD_600_ = 1, or diluted in 1X sterile PBS to either (B) 1/10, or (C) concentrated by resuspension in a volume ten times lower than the OD_600_ = 1 value. Mosquitoes were then anesthetized by a short (2–3 min) exposure to -20°C, and then injected with 69nL of bacteria or 1X PBS while prone on a chilled glass plate. Survival was then monitored daily for five days (S1 Fig). Based on this preliminary assessment, the 1/10 dilution produced approximately 10–25% mortality in *Ae. albopictus* by five days post-exposure for all bacteria, and was selected for use in subsequent immune priming experiments.

### Mortality assays

We exposed cohorts of adult female WT and Tet *Ae. albopictus* mosquitoes to low doses of one of the four pathogenic bacteria via intrathoracic injection, as above. In parallel, we ran two different control treatments: non-injected mosquitoes, which were anesthetized but not injected, and Mock challenged mosquitoes, which were injected with 1X sterile PBS, and served as a control for handling and injection quality. Mosquitoes were injected only during the afternoon hours (12pm – 5pm) to limit any effects of temporal differences in mosquito immune activity [69,70]. After recovery from injection and anesthesia, cohorts of 50 mosquitoes per treatment were placed in a cage and moved to a climate-controlled incubator (28°C, RH - 60–80%, photoperiod 12h: 12h) and provided with 10% sucrose via cotton pads, which were changed daily. To assess differences in mortality between treatments, the number of dead mosquitoes was scored daily either until all mosquitoes had died or until 50 days post-exposure. The assay was repeated three times, encompassing a total of 1800 mosquitoes.

The effects of *Wolbachia* infection, bacterial exposure treatment, and interactions between those variables on mosquito mortality were assessed using Cox Proportional Hazards Model with the coxme package in R [71]. Each of these terms satisfied the proportionality requirement of the analysis. The influence of experimental replicate was assessed as a random effect. The 95% confidence interval values for the coefficient of each term in the coxme model was calculated using the formula coefficient ± (1.96 x standard error), and these values, as well as the expected hazard ratio (*β*) were used to produce summary plots comparing hazards between treatments. The effect of *Wolbachia* on mosquito mortality for each treatment was estimated using pairwise comparisons (WT vs Tet) adjusted for multiple comparisons with Tukey’s test using the emmeans function in the R package of the same name.

### DENV vector competence assays

The DENV serotype-1 (strain BOLKW010, GenBank: JQ675358.1) used in this study was originally isolated from a DENV-infected patient in Key West, FL in 2010, and was provided by the Florida Department of Health Bureau of Public Health Laboratories. Propagation of DENV was conducted in kidney epithelial cells of African green monkey *Cercopithecus aethiops* (Vero E6, ATCC CRL-1586). Cells were cultured in M199 growth media (Cytiva HyClone, Logan, UT, USA), supplemented with 10% heat-inactivated fetal bovine serum, penicillin-streptomycin, and mycostatin, and were maintained at 37°C in an incubator with 5% CO_2_. The DENV stock was passaged in culture three times prior to the start of this study. Confluent monolayers of cells were inoculated with DENV at a multiplicity of infection of 0.01, and then incubated at 37°C with 5% CO_2_ for 1h. Next, 24 mL of M199 media was added to each 175 cm^2^ flask, which were then incubated for a further 7 days. Following the incubation period, virus-containing supernatant from these cells was harvested and used to challenge mosquitoes.

For vector competence assays, adult female WT and Tet mosquitoes aged between five and seven days post-eclosion were challenged with washed cells from the four bacterial stocks at OD_600_ = 1, diluted 1:10 in sterile 1X PBS, as described above. In parallel, cohorts of non-injected mosquitoes from both lines were included as handling controls, while cohorts of mock-injected mosquitoes received intrathoracic injections with sterile 1X PBS to gauge the mortality rate associated with the injection procedure. Five days later, surviving mosquitoes from all twelve cohorts were transferred to 16 oz cardboard cages (50 per cage) moved to the FMEL BSL-2 + /ACL-2 + laboratory, and starved overnight prior to oral feeding on DENV-infected blood. The five-day time point was selected to ensure sufficient numbers of mosquitoes would be likely to survive to the collection time in this assay (14 days post-DENV challenge) given the mortality rates observed in experiment one.

To prepare the infectious bloodmeal, defibrinated bovine blood (Hemostat Laboratories, Dixon, CA, USA) and adenosine triphosphate as a phagostimulant were combined with freshly harvested media from DENV-infected cells (6.1 log_10_ PFU/mL). Mosquitoes were offered a blood meal via Hemotek feeders (Discovery Workshops, Accrington, UK) pre-heated to 37°C for one hour. After this time, mosquitoes were anesthetized under CO_2_, and individuals that were fully engorged with blood were moved to new cages. Unfed or partially fed individuals were discarded. Cages were maintained in an incubator at 28°C with a 12:12‐h light: dark photoperiod regimen. During this time, mosquitoes were fed on 10% sucrose provided via cotton pads, which were changed daily.

Mosquitoes were dissected at 14-days post infection (dpi), a commonly used diagnostic time point for assessing DENV infection and disseminated infection [32]. Legs and wings were removed from each mosquito using sterile forceps. This interval was selected to approximate, or exceed, the extrinsic incubation period (EIP), thereby ensuring sufficient time for viral dissemination beyond the midgut and permitting the opportunity for infection of the salivary glands. At 28°C, the EIP for DENV is approximately 10 days, based on Bayesian time-to-event models synthesized from multiple published datasets [72]. Mosquito saliva, as a proxy for DENV transmission, was collected from these specimens by forced salivation using microhematocrit capillary tubes containing type B immersion oil (Cargille Laboratories, Cedar Grove, NJ, USA). The proboscis of each mosquito was inserted in a capillary tube, then mosquitoes were left to salivate for 1 h. After this time, the contents of each capillary were independently deposited in a microcentrifuge tube containing 300 µL of M199 media. Body samples (thorax and abdomen) and legs from these specimens were placed separately in microcentrifuge tubes containing 1 mL of M199 media. All mosquito samples were stored at -80°C until further processing. This assay was repeated three times using independent cohorts of mosquitoes.

### DENV detection and quantification

The presence and viral titer of DENV infectious particles in mosquito tissues were determined using plaque forming assays with Vero cells. The day prior to the assays, 12-well plates were seeded with Vero cells (1.5x10^5^ cell/mL) and maintained with M199 media supplemented with 10% heat-inactivated fetal bovine serum, 2% penicillin-streptomycin, and 0.2% mycostatin, at 37°C and a 5% CO_2_ atmosphere. To account for high mortality amongst mosquito cohorts that were exposed to pathogenic bacterial infection, a random subset of 20 specimens per treatment (5–8 specimens per replicate) were selected from across the three replicate vector competence assays. Body, leg, and saliva samples from these specimens were homogenized at 19.5 Hz for 3 min, and then centrifuged at 13,200 rpm for 5 min. Samples were then subjected to 10-fold serial dilutions in M199 media and then 200μL of diluent was inoculated onto cell monolayers. Plates were then incubated at 37°C and a 5% CO_2_ atmosphere for 1h and rocked every 15 min to allow for dispersal across the monolayer and viral adsorption to cells. After this interval, an immobilizing overlay of 1.5mL of 1% methylcellulose was applied to each well to restrict DENV growth to the originally infected foci of cells. Cells were then incubated for 7 days, after which the methylcellulose overlays were removed, and cells were stained with 1mL of crystal violet basic red solution (0.25% hexamethyl pararosaniline chloride) per well to enhance visualization and enumeration of DENV plaques. Stained plates were rinsed with tap water, dried, and scored for cytopathic effects.

Each well was classified as DENV-positive or DENV-negative based on the presence or absence of cytolytic plaques, respectively. DENV loads were calculated across five dilutions per sample (10^-1^ to 10^-5^), with values expressed as plaque forming units per milliliter (PFU/mL). Susceptibility to DENV infection, dissemination, and transmission were determined by the presence of DENV infectious particles in the mosquito body, legs, and saliva, respectively. DENV prevalence and viral load were independently examined in the bodies, legs, or saliva of each mosquito. Legs and saliva from specimens where bodies were negative for DENV were not subject to further testing. Likewise, the saliva of specimens where bodies were positive, but legs were negative for DENV, were not tested due to the lack of a disseminated infection.

DENV prevalence was compared between treatments using generalized linear regression with a binomial distribution using the glm function in R. Terms in the model included: (1) *Wolbachia* infection status, (2) whether mosquitoes had been primed with a gram positive bacterium, gram negative bacterium, or were not exposed to bacteria, and (3) interaction between *Wolbachia* and priming effect. DENV load was compared between treatments for samples positive for the virus using generalized linear regression with a gaussian distribution. These models included the same terms as the DENV prevalence models, and were run using the glm function in R. Initial versions of models were run using a bacterial treatment effect term (independently assessing the impact of each bacterial isolate), however, this term was consistently less informative than the priming effect term, and was therefore excluded from the final models. Additionally, replicate was not a significant predictor of DENV prevalence or DENV load and was excluded from the final statistical models.

### Software

Data were analyzed using R version 4.5.1 and GraphPad prism for macOS version 11.0.1 (99). Figure panels were produced using GraphPad prism and were assembled in Microsoft PowerPoint for Mac version 16.103.1. All R scripts are provided in S2 File.

## Results

### Effects of Wolbachia on bacteria-mediated mortality

In response to intrathoracic challenge with four different mosquito-associated bacterial isolates, we observed delayed mortality associated with native *Wolbachia* infection in *Ae. albopictus* mosquitoes (Fig 1A-Fig 1F). Analysis of data from all treatments (S3 File) via Cox Proportional Hazards Model (CPH) revealed a significant increase in risk associated with the absence of *Wolbachia* (CPHL: *β* = 1.391, *Z* = 2.31, *P* = 0.0210). After bacterial challenge, median survival times were consistently 6–8 days shorter for *Wolbachia*-free Tet mosquitoes compared to *Wolbachia*-infected WT mosquitoes. Furthermore, pairwise comparisons of mortality profiles between WT and Tet mosquitoes revealed significantly increased mortality in the absence of *Wolbachia* after challenge with *Micrococcus* (Tukey’s test: *Z =* -6.681, *P* < 0.0001), *Staphylococcus* (*Z =* -5.885, *P* < 0.0001), *Brevundimonas* (*Z =* -4.815, *P* < 0.0001), and *Enterobacter* (*Z =* -4.889, *P* < 0.0001). These results indicate that *Wolbachia*-mediated protection against bacteria-induced mortality occurred in response to challenge with each of the four bacteria.

**Fig 1:**
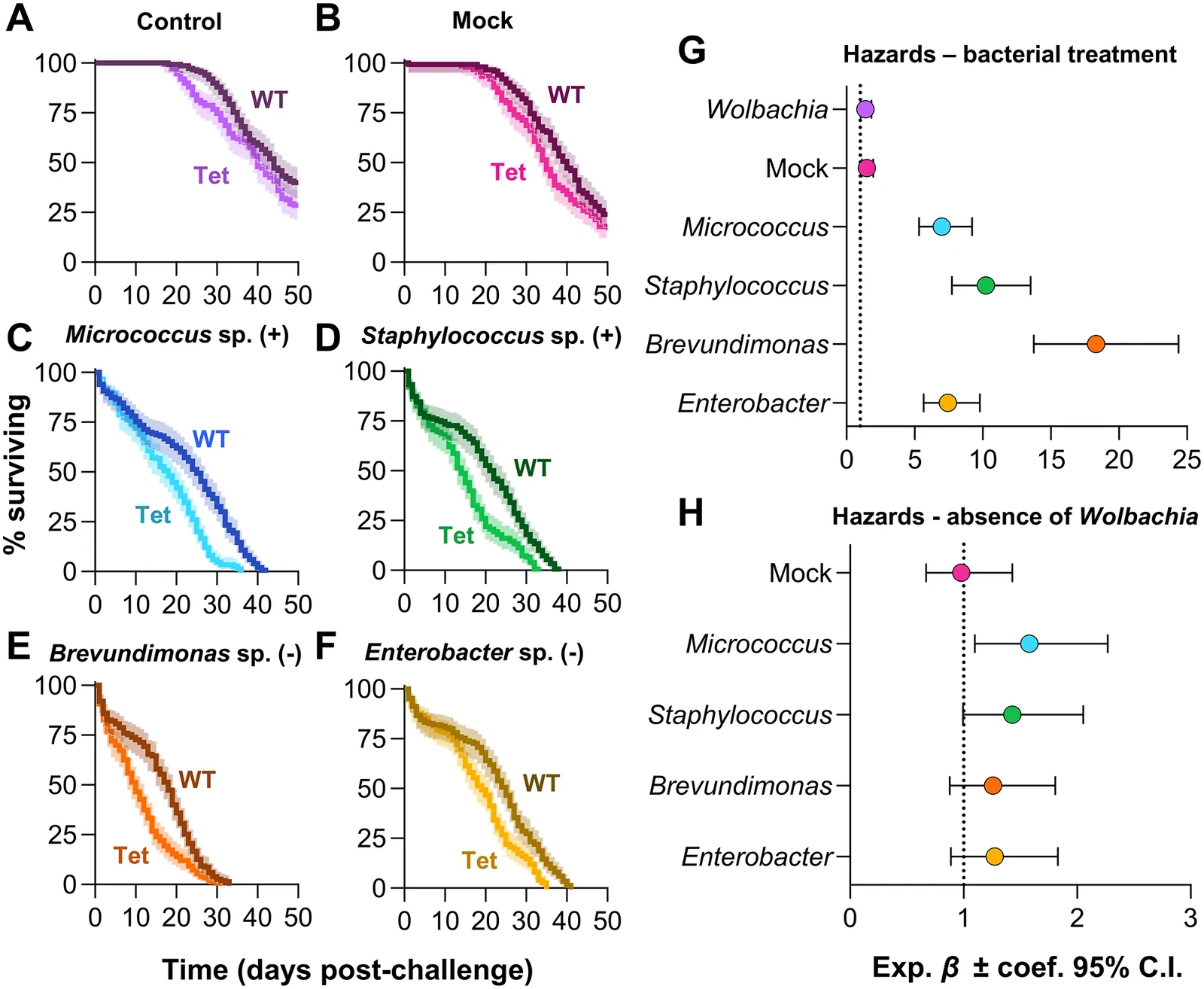
*Wolbachia* infection protects *Aedes albopictus* mosquitoes from bacteria-induced mortality. Mortality was compared between *Wolbachia*-infected (WT) and -uninfected (Tet) *Ae. albopictus* after sub-lethal intrathoracic challenge with bacteria. Mortality was assessed daily for 50 days in cohorts of 50 adult, female *Ae. albopictus* from one of six treatments: non-injected controls **(A)**, Mock challenged with sterile 1X PBS **(B)**, challenged with *Micrococcus* sp. **(C)**, or *Staphylococcus* sp. **(D)** (gram positive), or with *Brevundimonas* sp. **(E)**, or *Enterobacter* sp. **(F)** (gram negative). Plus (+) and minus (-) signs in bacterial treatment names reflect their gram status. *Wolbachia* infection significantly reduced mortality after challenge with all four bacteria as determined by assessment with Cox Proportional Hazards Model. Significant risk of mortality was observed after challenge with any bacteria (*β* = 7.0 – 18.3) as indicated by plots of the hazard ratio (*β*, ± 95% confidence interval) **(G)**. Values depict the expected risk associated with each treatment, regardless of *Wolbachia* infection status. *β* = 1 indicates no difference in risk from the WT control line. Hazards associated with bacterial challenge were higher in the absence of *Wolbachia* infection **(H)**, with a significant interaction observed for *Wolbachia* and *Micrococcus* (*β* = 1.579, *Z* = 2.47, *P* = 0.0137) and a marginal effect observed for *Wolbachia* and Staphylococcus (*β* = 1.259, *Z* = 1.94, *P* = 0.0528). Values indicate expected risk associated with each treatment for Tet mosquitoes relative to WT. These data were derived from three independent experiments, incorporating 1800 individual mosquitoes.

Comparison of all treatments revealed that significantly increased risk was associated with each of the four bacterial treatments (CPH: all *P* < 2e-16), indicating that *Ae. albopictus* mortality increased in response to bacterial challenge. Expected hazard ratios varied depending on the bacterial isolate, with *Brevundimonas* associated with the greatest risk (*Micrococcus* - *β* = 7.006; *Staphylococcus* - *β* = 10.230; *Brevundimonas* - *β* = 18.312; and *Enterobacter* - *β* = 7.440) (Fig 1G). We also assessed differences in pathogen blocking between bacterial isolates by considering *Wolbachia* by bacterial treatment interaction terms (Fig 1H)**.** Hazards associated with bacterial challenge in the absence of *Wolbachia* were between 1.26 and 1.58 times higher, indicating the provisioning of moderate protection by *Wolbachia*. This effect was stronger for the two gram positive bacteria compared to the two gram negative bacteria, with a significant interaction observed for *Wolbachia* and *Micrococcus* (CPH; *β* = 1.579, *Z* = 2.47, *P* = 0.0137), and a marginal but non-significant interaction for *Wolbachia* and *Staphylococcus* (*β* = 1.430, *Z* = 1.94, *P* = 0.0528). Smaller, non-significant interactions were observed for *Wolbachia* and *Brevundimonas* – (*β* = 1.259, *Z* = 1.25, *P* = 0.2114), and for *Wolbachia* and *Enterobacter* – (*β* = 1.274, *Z* = 1.31, *P* = 0.1897). Overall, these results suggest that *Wolbachia*-mediated protection against bacterial induced mortality was more effective against the two gram positive bacteria.

There were two confounding factors in this assay. First, we observed lower base fitness in the Tet line compared to the WT line, equating to a four-day difference in median mortality between the two unexposed control lines. Secondly, we observed a modest, but significant increase in hazard associated with mock injections, with median mortality in those treatments four days shorter than compared to non-injected controls (CPH: *β* = 1.488, *Z* = 2.82, *P* = 0.0049). However, pairwise comparisons for both of those treatments, revealed no significant difference in mortality associated with *Wolbachia* infection status (Tukey’s test: Non-injected - *Z =* -2.308, *P* = 0.4709; Mock - *Z =* -2.371, *P* = 0.4260).

### Effects of Wolbachia and bacterial priming on dengue infection

To assess the impact of *Wolbachia* infection and bacterial challenge on DENV infection, we challenged WT and Tet *Ae. albopictus* with a sub-lethal dose of one of four bacterial isolates. Six days later, surviving mosquitoes were orally challenged with DENV-1 BOLKW010. Comparisons of DENV prevalence in *Ae. albopictus* bodies at 14 dpi (Fig 2A, S4 File) revealed no significant effect of *Wolbachia* infection (GLM: *Z* = 0.353, *P* = 0.72394), indicating that the native *Wolbachia* infections did not influence susceptibility to DENV infection. However, assessment of the impact of bacterial treatment revealed that pre-challenge with one of the two gram negative bacterium resulted in a relative reduction in DENV prevalence of 23.61% compared to unexposed mosquitoes (GLM: *Z* = -2.671, *P* = 0.0076). No change in prevalence was observed after pre-challenge with one of the two gram positive bacterial isolates (GLM: *Z* = -0.624, *P* = 0.5328). Similarly, there was no significant interaction between bacterial priming and *Wolbachia* infection (GLM: Gram positive - *Z* = 0.151, *P* = 0.8799; Gram negative - *Z* = 0.668, *P* = 0.5041), suggesting that the observed decrease in DENV prevalence after *Brevundimonas* or *Enterobacter* challenge was not linked to *Wolbachia*-mediated pathogen blocking. Interestingly, comparisons of DENV prevalence in legs (Fig 2B) and saliva (S2 Fig) samples, also collected at 14dpi, revealed no significant effects due to *Wolbachia* infection or bacterial priming (GLM: *P* > 0.05). This suggested that the priming effect seen with the two gram negative bacterial isolates did not impact the rate of disseminated DENV infection and saliva infection.

**Fig 2:**
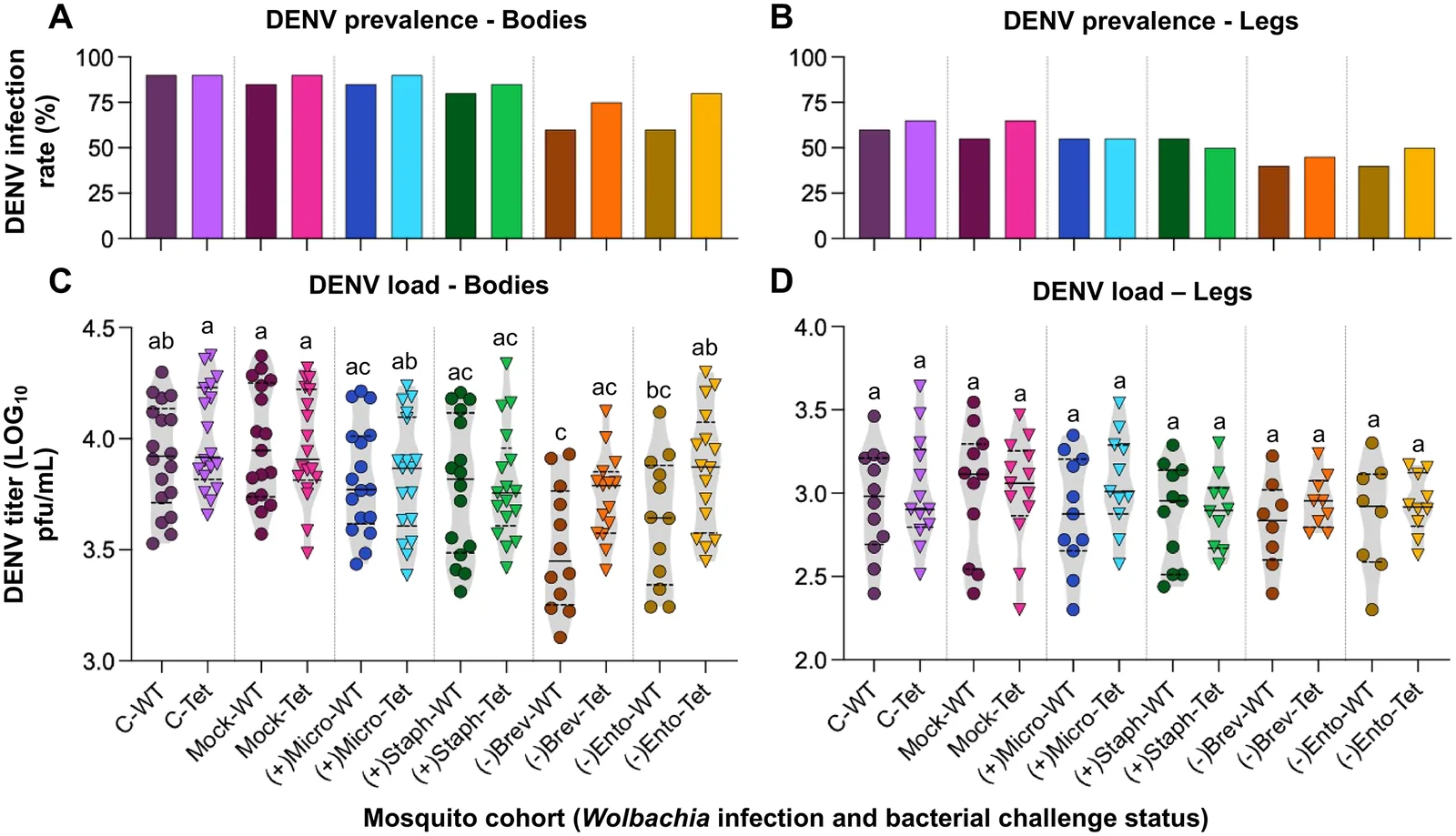
Bacterial priming and *Wolbachia*-mediated DENV blocking in *Aedes albopictus* after systemic bacterial challenge. *Wolbachia*-infected (WT) and -uninfected (Tet) *Ae. albopictus* were pre-challenged with a sub-lethal dose of *Micrococcus* sp. (Micro), *Staphylococcus* sp. (Staph), *Brevundimonas* sp. (Brev), or *Enterobacter* sp. (Ento) and then orally challenged with DENV 6 days later. At 14 days post-DENV challenge, DENV prevalence was characterized in mosquito bodies **(A)** and legs **(B)**, as a proxy for disseminated infection, using plaque-forming assays. Bars depict the percentage of mosquito specimens where DENV plaques were observed, by treatment. Unexposed (C) and Mock-injected (Mock) control lines were run to compare the impacts of bacterial challenge and injection. In bodies, pre-challenge with either of the two gram negative bacteria led to a significant reduction in DENV prevalence (GLM: *Z* = -2.671, *P* = 0.0076), however no significant effects were observed due to infection with *Wolbachia* or challenge with one of the two gram positive bacterial isolates. No significant effects on DENV prevalence were observed in legs. DENV load was assessed in mosquito bodies **(C)** and legs **(D)** at 14 dpi. Violin dot plots depict the LOG_10_ DENV titer in plaque-forming units per mosquito specimen. DENV load in *Ae. albopictus* bodies was significantly reduced by pre-challenge with bacteria (GLM: *P* < 0.05), but not due to *Wolbachia* infection (GLM: *P* = 0.5337). However, pre-challenge with a gram negative bacterium reduced DENV load in a *Wolbachia*-specific manner (GLM: *t* = 2.108, *P* = 0.0363). No significan*t* effects on DENV load were observed in legs. Dashed lines depict treatment medians. Dotted lines depict treatment 25th and 75th percentile quartile values. Letters above each plot depict statistical differences between treatments as determined by Tukey’s test. Plus (+) and minus (-) signs in bacterial treatment names reflect their gram status. Data were collected from 20 body and leg samples per treatment, from 240 mosquito specimens collected across three independent experiments.

DENV load in *Ae. albopictus* bodies (Fig 2C) was significantly reduced by priming with one of the gram positive isolates (GLM: *t* = -2.313, *P* = 0.0218) or one of the gram negative bacterial isolates (GLM: *Z* = -5.569, *P* = 8.77e-8). Conversely, it was not impacted by the presence or absence of *Wolbachia* (GLM: *t* = 0.624, *P* = 0.5337). These effects indicated that there was an impact of bacterial priming but no overall impact of *Wolbachia*-mediated pathogen blocking on DENV load. However, assessment of interaction between bacterial challenge and *Wolbachia* infection revealed that pre-challenge with either the *Brevundimonas* or *Enterobacter* isolates in the presence of *Wolbachia* resulted in significantly lower DENV loads (GLM: *t* = 2.108, *P* = 0.0363). No such interactive effects were seen between *Wolbachia* and either gram positive bacterium (GLM: *t* = -0.322, *P* = 0.7479). As with DENV prevalence, no effects of *Wolbachia* infection or bacterial priming were observed for DENV load in legs (Fig 2D) or saliva samples (S2 Fig), which suggested that the effects did not carry over to other tissues.

## Discussion

### Wolbachia-mediated bacterial protection in Aedes albopictus

We have identified several bipartite and tripartite microbial interactions that shape *Ae. albopictus* responses to pathogen infection. Native *Wolbachia* markedly enhances the ability of *Ae. albopictus* to survive systemic infection by four different bacteria (two gram positive and two gram negative), confirming that bacterial pathogen blocking occurs in mosquitoes harboring native *Wolbachia*. Notably, protection was significantly stronger against the two gram positive bacteria, consistent with heightened Toll pathway activation by *Wolbachia* [36,37].

This pattern is not consistent with what has been seen in all other *Wolbachia*-host systems. In *D. melanogaster*, native *Wolbachia* does not protect against bacteria-induced mortality following intrathoracic challenge [28–30]. However, blocking has been observed after oral exposure to pathogenic bacteria [28], and intrathoracic challenge with multiple fungal species [31]. These findings suggest fundamental differences in how native *Wolbachia* mediate antibacterial protection across different insect clades, although further studies are needed to validate this hypothesis.

Antibacterial effects also vary amongst transinfected *Ae. aegypti* [22,27]. The *w*MelPop strain confers stronger protection than *w*Mel, reducing bacterial load and delaying mortality across all four bacterial species examined. For *w*Mel, those two effects were only observed after *Erwinia caritivora* challenge. For *Burkholderia cepacia*, only reduced pathogen load was observed, while for *Salmonella typhimurium* only reduced mortality was observed. In contrast to our observations, no blocking was observed against *Mycobacterium marinum*, the lone gram positive bacterium that was examined [27]. These patterns align with the broader observation that *w*Mel infection is associated with lower *Wolbachia* density, more modest pathogen blocking, and less extensive manipulation of host immunity relative to *w*MelPop [18,37]. Overall, these findings highlight that there are broad differences in the antibacterial capacity of *Wolbachia* across different insect systems. Based on our experimental design, it was not possible to determine whether the decrease in risk associated with *Wolbachia* infection was due to an enhanced ability to clear bacterial infection, or increased tolerance to that infection. Accordingly, there is a need for a more rigorous examination of the putative influences on this phenotype to understand the contributions of host, pathogenic bacterial isolate, initial dosage, changes in bacterial load over time, pathogen delivery method and *Wolbachia* strain.

### Bacterial priming limits DENV infection in Aedes albopictus in the absence of Wolbachia-mediated DENV blocking

In our second experiment, we observed priming effects resulting from bacterial challenge, irrespective of host *Wolbachia* infection status. Priming with one of the two gram positive bacterial isolates resulted in reduced DENV load, whereas priming one of the two gram negative bacteria had a broader impact, lowering both DENV prevalence and viral load. This differential is notable given that gram negative bacterial infections predominantly activate the IMD pathway [73], which is generally considered less important to the mosquito response to dengue infection than the gram positive bacteria-responsive Toll pathway [74]. Crosstalk between these two pathways is well documented [75,76] and such interactions may underlie the enhanced antiviral effects observed here. Our findings are reflective of a typical immune priming response in insects, although we were not able to verify whether an immune response was induced in response to *Wolbachia* infection, bacterial challenge, or DENV challenge in our experimental system. It should also be noted that even with sub-lethal exposure to the four bacteria, we did observe mortality. This indicates that the pool of mosquitoes used for DENV exposure that had survived bacterial challenge for six days, may have represented a non-random subset of mosquitoes with greater robustness or immune activity. Immune priming has been observed in several mosquito-pathogen systems, including in *Ae. aegypti* with DENV, where reduced DENV competence occurred following priming with inactivated DENV [77], and altered DENV load occurred after larval challenge with *Enterobacter ludwigii* [51]. Meanwhile, in *Anopheles gambiae*, an initial challenge with *Escherichia coli* facilitated a greater ability to clear a subsequent infection with a different *E. coli* strain [78].

We observed no evidence of *Wolbachia*-mediated DENV blocking in either non-injected control or mock-injected mosquitoes, based on both viral prevalence and viral load metrics. Based on previous studies, arbovirus blocking in *Ae. albopictus* is either weak [14,79] or absent [59]. Our results are in keeping with these findings, as well as examinations of native infections in *Aedes fluviatilis* [80], and *Aedes notoscriptus* [81] that did not observe any DENV blocking. These results contrast sharply with the strong and consistent antiviral effects of the *w*Pip *Wolbachia* strain in the *Culex pipiens* species complex against West Nile virus [15,16,82], as well as the robust antibacterial protection observed in our bacterial challenge experiment. Collectively, these findings confirm the inherent variability in pathogen blocking amongst mosquitoes harboring native *Wolbachia* infections, and underscore the need to determine the relative contribution of host background, bacterial isolate, infectious dose, pathogen delivery route, and *Wolbachia* strain.

### Priming with two Gram negative bacteria prompts slightly stronger Wolbachia-mediated DENV blocking

Our finding that pre-challenge with either *Brevundimonas* or *Enterobacter* significantly reduced DENV titers in the bodies of *Wolbachia*-infected *Ae. albopictus* yields two important insights. Firstly, it indicates that bacterial priming and *Wolbachia*-mediated pathogen blocking can interact within a native host background, with the effect potentially driven by gram negative bacteria. However, we note that there is a need to examine additional bacteria in this experimental system, as we have only tested two of each gram status. It is possible that different effects on *Wolbachia-*DENV dynamics may result from exposure to different bacteria, regardless of their gram status. Secondly, it demonstrates latent, inducible antiviral potential in native *Wolbachia* infections, even though the magnitude of these effects is modest and currently limited to body tissues, raising the questions about their broader applicability to virus transmission. A similar interaction between bacterial priming and *Wolbachia* has been observed in the isopod *A. vulgare*, where priming and non-lethal *Salmonella enterica* enhanced survival after a subsequent high-dose challenge in *Wolbachia*-infected hosts [58]. The mechanistic basis of these interactions remains unclear. Immune priming has been linked to the induction of innate immune pathways leading to increased production of antimicrobial peptides [83]. It also involves cellular immune processes including the Lipoxin/Lipocalin complex, which promotes hemocyte differentiation [84], and prostaglandin E2, which promotes hemocyte attraction [85].

*Wolbachia*-mediated pathogen blocking has been associated with activation of innate immunity, with broad upregulation of immune effectors [22,36,37,86], although its influence on mosquito cellular immunity is less well characterized. *Wolbachia* can be found in its host’s hemocytes, as in *A. vulgare* [87]. However, a study in *D. melanogaster* found no evidence of *Wolbachia* infection altering host phagocytic activity [88]. The bacterium does induce high levels of reactive oxygen species in *Ae. aegypti* [36], and *D. melanogaster* [89], which contributes to virus blocking. Native *Wolbachia* infection in *D. melanogaster* and transinfection in *Ae. aegypti* are both associated with increased melanization in host hemolymph [44]. In *Ae. aegypti*, infection is also associated with upregulation of genes in the melanization pathways, including a prophenoloxidase [37].

Whether native *Wolbachia* infection in *Ae. albopictus* induces a cellular and/or humoral immune response remain unresolved, as the molecular consequences of infection have not been characterized in this mosquito. Consequently, the effects we observed in this study may have been caused by still-uncharacterized *Wolbachia*-mediated impacts on physiological processes including immunity, physiology, microbiome composition, or metabolic status, amongst others. The transcriptomic analyses of *Ae. fluviatilis* with and without its native *Wolbachia* strain offer potential insight. *Wolbachia* infection in *Ae. fluviatilis* had a small impact on immune response, increasing levels of two pathogen-binding and two bacterial cell wall degrading proteins, and modulating the oxidative stress response [49]. Further studies revealed that infection was associated with higher levels of triglycerides and glycogen after blood feeding [90], and higher levels of ROS in midguts [80]. These findings show that there is some latent stimulation of immune processes with native infections even in the absence of arboviral blocking. Similar processes could underlie the interaction that we observed in *Ae. albopictus*. Heightened immune activity associated with *Wolbachia* infection [17], pathogen blocking [60], and immune priming [91,92] are all known to be metabolically and physiologically costly for insects. Further investigation is warranted to find out whether and how interactions between immune priming and pathogen blocking occur, and to elucidate any broader impacts on host biology.

### Study limitations

Our study has several limitations that merit discussion. Firstly, our results were obtained for a single *Ae. albopictus* population. It is unclear whether similar responses would occur across other genetic backgrounds or other mosquito species that harbor native *Wolbachia* infections. Likewise, we have performed these experiments using a modest number of bacterial isolates, a single DENV isolate, all used at a single dosage. Broader exploration is required to determine whether the effects we have observed here are consistent across a greater range of microorganisms, incorporating challenges with different bacteria, fungi, and other arboviruses, potentially incorporating specific quantification of *Wolbachia* and each bacterium, as changing microbial titers may have contributed to our results. Similarly, our findings have not been examined in *Wolbachia* transinfected mosquitoes, which have a broad importance to arbovirus transmission given their role in large-scale mosquito control projects [26,93]. We observed higher baseline fitness associated with *Wolbachia* infection in the form of greater longevity. This implies that comparison of the two populations is not made from the same baseline. However, fitness costs associated with curing a native *Wolbachia* infection are common and potentially reflect the mutualistic nature of these host-symbiont relationships [3,15,94,95] in comparison to transinfections, where high fitness costs are typically associated with infection [96]. It is possible that injected *Ae. albopictus* had not cleared the bacterial infection at the time that they were challenged with DENV, which means that findings in that experiment may have reflected the consequences of bacterial-viral co-infection. Further investigation of these microbe-microbe interactions over time would be an interesting future research direction. We also observed a significant mortality effect associated with injection, which is not desirable in a microbial challenge experiment. However, this effect was measured across a 50-day post-challenge period, which is longer than the conventional monitoring period [9,10]. We also observed that the scale of the mortality cost associated with injection was not different between the WT and Tet lines, indicating that it was unlikely to have a major impact on the interpretation of post-bacterial challenge survival data. Finally, due to the scope of the research here, we are not able to propose a mechanism for putative interactions between bacterial priming and *Wolbachia*-mediated pathogen blocking, which is an important future research direction.

### Conclusion

Through challenge of *Wolbachia* infected and -uninfected mosquitoes with four pathogenic bacterial isolates, we demonstrated that the native *Wolbachia* of *Ae. albopictus* consistently protect their host from bacteria-induced mortality. We also demonstrate that pre-challenge with these bacteria reduces the burden of DENV infection in their host. Interestingly, we observed contrasting interactions between *Wolbachia* and pathogenic bacteria depending on their gram status. Challenge with gram positive bacteria was associated with stronger *Wolbachia*-mediated protection against mortality. Meanwhile, pre-challenge with gram negative bacteria was associated with more effective *Wolbachia*-mediated DENV blocking. These interactions highlight the complexity of microbial interactions associated with native *Wolbachia* infections and their contribution to the immune response of a prominent mosquito vector.

## Supporting information

S1 File Bacterial Sequences. This file contains the raw sanger sequencing output for the four bacterial isolates used in this study.
(XLSX)

S2 File R Scripts. All R scripts used to complete the analysis of data in this study.
(DOCX)

S3 File Survival assay data. Raw and processed data associated with the survival assay. Includes statistical output.
(XLSX)

S4 File Vector competence assay data. Raw and processed data associated with the dengue vector competence assay. Includes statistical output.
(XLSX)

S1 Fig Dose-response mortality curves for *Aedes albopictus* mosquitoes challenged with bacteria via intrathoracic injection. Mortality was assessed for *Wolbachia*-infected (WT) *Ae. albopictus* after sub-lethal intrathoracic challenge with *Micrococcus* sp. **(A)**, or *Staphylococcus* sp. **(B)** (gram positive, +), or with *Brevundimonas* sp. **(C)**, or *Enterobacter* sp. **(D)** (gram negative, -). Adult female mosquitoes were challenged with one of three doses of bacterial cells washed in sterile 1X PBS and diluted (0.1x) or concentrated (10x) from an OD_600_ = 1 by adjusting the final volume. To control for mortality due to injection, mosquito cohorts were also challenged with 1x PBS (Control). Mortality was recorded daily for five days post-challenge. Graphs depict the daily survival rate ± 95% confidence interval. Data represent 17–27 mosquitoes per treatment from two replicate experiments, for a total of 608 specimens.
(TIF)

S2 Fig DENV prevalence and load in *Aedes albopictus* saliva after systemic bacterial challenge. *Wolbachia*-infected (WT) and -uninfected (Tet) *Ae. albopictus* were pre-challenged with a sub-lethal dose of *Micrococcus* sp. (Micro), *Staphylococcus* sp. (Staph), *Brevundimonas* sp. (Brev), or *Enterobacter* sp. (Ento) and then orally challenged with DENV 6 days later. At 14 days post-DENV challenge, DENV prevalence was characterized in mosquito saliva **(A)** as a proxy for the DENV transmission rate, using plaque-forming assays. Bars depict the percentage of mosquito specimens where DENV plaques were observed, by treatment. Unexposed (C) and Mock-injected (Mock) control lines were run to compare the impacts of bacterial challenge and injection. No significant effects were observed due to *Wolbachia* or bacterial challenge. DENV load was also assessed in mosquito saliva **(B)** at 14 dpi. Violin dot plots depict the LOG_10_ DENV titer in plaque-forming units per saliva specimen. No significant effects were observed due to *Wolbachia* or bacterial challenge. Plus (+) and minus (-) signs in bacterial treatment names reflect their gram status. Solid horizontal lines depict treatment medians. Dashed lines depict treatment 25 and 75 percentile quartile values. Letters above each plot depict statistical differences between treatments as determined by Tukey’s test. Data were collected from 20 body and leg samples per treatment, from 240 mosquito specimens collected across three independent experiments.
(TIF)
