# Supplementary material for: Bacterial immune priming and Wolbachia-mediated dengue blocking in Aedes albopictus mosquitoes
Source: PLoS Negl Trop Dis. 2026 Oct 16;20(10):e0014807. doi: 10.1371/journal.pntd.0014807 (PMC13712725; doi:10.1371/journal.pntd.0014807)
Supplement: Supplementary file 1 [file pntd.0014807.s002.docx]

**S2 File: R Scripts**

install.packages("ggcorrplot")

install.packages(c("survival", "survminer"))

install.packages("coxme")

install.packages("emmeans")

library(ggcorrplot)

library(survival)

library(survminer)

library(coxme)

library(emmeans)

**A. Survival post-bacterial challenge**

#coxme with treatment#

cox_me <- coxme(Surv(Time, Event) ~ Wolbachia*Treatment + (1 | Rep), data = Mortality_data)

summary(cox_me)

exoprop_coxme <- cox.zph(cox_me)

prop_coxme

##compare between paired WT/Tet treatments to gauge the scale of pathogen blocking##

\# Estimate marginal means for treatment#

fit_emm <- emmeans(cox_me, specs = pairwise ~ Wolbachia:Treatment, type = "response")

fit_emm

**B. DENV prevalence in bodies, legs, and saliva**

##Binomial glms##

##glm - bodies##

prev_body_glm <- glm(formula = DENV ~ Wolbachia*Gram, family = binomial, data = Prev_bodies)

prev_body_glm

summary(prev_body_glm)

##glm - legs##

prev_legs_glm <- glm(formula = DENV ~ Wolbachia*Gram, family = binomial, data = Prev_legs)

prev_legs_glm

summary(prev_legs_glm)

##glm - saliva##

prev_saliva_glm <- glm(formula = DENV ~ Wolbachia*Gram, family = binomial, data = Prev_saliva)

prev_saliva_glm

summary(prev_saliva_glm)

**C. DENV load** **in bodies, legs, and saliva**

##glm - bodies##

body_glm <- glm(formula = Viral_load ~ Wolbachia*Gram, family = gaussian, data = Load_bodies)

body_glm

summary(body_glm)

##glm - legs##

legs_glm <- glm(formula = Viral_load ~ Wolbachia*Gram, family = gaussian, data = Load_legs)

legs_glm

summary(legs_glm)

##glm - saliva##

saliva_glm <- glm(formula = Viral_load ~ Wolbachia*Gram, family = gaussian, data = Load_saliva)

saliva_glm

summary(saliva_glm)
