# Supplementary material for: Bacterial immune priming and Wolbachia-mediated dengue blocking in Aedes albopictus mosquitoes
Source: PLoS Negl Trop Dis. 2026 Oct 16;20(10):e0014807. doi: 10.1371/journal.pntd.0014807 (PMC13712725; doi:10.1371/journal.pntd.0014807)
Supplement: Supplementary file 2 [file pntd.0014807.s007.docx]

We wish to thank all of the reviewers for their helpful comments on the manuscript. We believe that the revised version is much improved and also adequately addresses all of the issues that were raised during review.

Reviewer #1: This manuscript by Alomar et al. assesses whether bacterial immune priming in Aedes mosquitoes results in any measurable impact on Wolbachia’s interactions with dengue virus in the host. Here, the authors use several bacterial pathogens isolated from mosquitoes and demonstrate that low-dose infections can measurably increase protection against subsequent dengue virus when considering viral load. This is moderately enhanced by the Wolbachia symbiont. The strength is in the experimental design and analysis, which is rigorous. The weaknesses, acknowledged by the authors, are that the study is limited in scope and that the phenotype/effects measured are fairly moderate in strength. However, it is beneficial, as immune priming is poorly understood in the context of Wolbachia. The paper is straightforward, with good experimental design, and demonstrates results from a couple of key experiments.

A few general questions/comments for the authors:

• Immune priming:

o Is it known if this mosquito typically clears bacterial infection within the timeline in this study?

This is unclear and we have mentioned this point as an additional caveat in the study limitations section (lines 658-662)

o L501: careful with wording in cases like this- “resistance” might be misread as immune resistance which was not tested here

We have edited the text in a few places (lines 26-27, 146-149, 518-520) to avoid use of this word and to more accurately describe the effects of both bacterial priming and Wolbachia-mediated pathogen blocking observed in our study.

o It may help to discuss hypotheses other than immune priming here since the priming has not been more directly tested. For example, it may have to do with altered physiology or microbiome or nutrient status post-bacterial infection that then impacts dengue virus. Sometimes the wording in the manuscript makes it seem like immune priming is the definite mechanism here, which it is likely to be, but not yet proven. Since it is in the title/abstract etc., I would suggest some rewording and further discussion (maybe “consistent with immune priming” or “immune priming-like”, etc.). I thought the wording of “bacterial priming” that was often used was a good alternative.

Thank you for the suggestion. We have modified the text in line with this comment. First, we have acknowledged that we have not verified whether an immune response was induced (lines 561-568). We then discuss some of the alternative hypotheses you propose in your comment in the discussion (lines 622-625). Finally, we have edited the text so that the interpretation of our results being due to immune priming is less definitive, using your suggested wording of “bacterial priming”.

• Figures 1&2: include gram status in figure for clarity- the text refers to effects of gram positive vs negative microbes, but the reader has to reference the text and go back and forth to the figure. It would be better to have the gram status labelled on the figure to compare to the conclusions in the text.

We have added this information to these figures and also the two supplementary figures. Figure legends have been updated to reflect this change.

• Fig 2C/D: The statistical bars (median, etc.) are difficult to see. Please modify so that they lie on top of the dots. Same for the S2B plot.

We have moved the median and quartile lines from underneath to above the data points. We have also changed the line pattern for medians and quartiles to make them more easily visualized.

Reviewer #2: The study by Alomar et al. asks an interesting question whether native Wolbachia provides anti-bacterial pathogen protection and whether bacterial immune priming interacts with native Wolbachia-conferred pathogen blocking in Ae. Albopictus. The experiments are generally well executed. Authors did a good job acknowledging the limitations of their assays. However, the central claim that revolves around immune priming is not fully supported by the presented data. See my comments below:

Major comments:

1. My biggest concern is that "Immune priming" is assumed rather than demonstrated in the study. Immune priming is more than exposing insects to bacteria before another infection. Authors exposed mosquitoes to a sublethal bacterial infection followed by DENV challenge five days later, but they do not establish whether the initial bacterial infections had been cleared before viral exposure. Consequently, the observed reduction in viral prevalence or load could equally reflect persistent bacterial infection, chronic immune activation, altered metabolism, or physiological stress rather than an induced memory-like immune state. Throughout the manuscript, the authors interpret these effects as immune priming without directly demonstrating that a primed immune state exists. They could have measured bacterial loads immediately before and 5 days after injection. If bacteria persist 5 days after injection, the subsequent viral challenge would represent a simultaneous bacterial-viral co-infection rather than an immune priming experiment. Making this distinction is critical for interpretation of the results.

We agree with this statement and note that similar issues were raised by Reviewer #1. We have revised the text, most notably in the discussion, to highlight potential alternative causes of our findings, rather than immune priming/immune memory. This includes the stressors you have listed above. We have also clarified that it is not certain whether the mosquitoes in our DENV vector infection experiment had cleared their initial bacterial infection at the time of viral challenge. In line with these changes, we have also tempered language in the manuscript relating to immune priming and its potential role in our findings.

2. The authors show that Wolbachia-infected mosquitoes survive longer after bacterial challenge, but it remains unclear whether this reflects enhanced bacterial clearance (resistance) or increased tolerance to bacterial infection. Since theirs is the first study measuring native Wolbachia effect in Ae. Albopictus, measuring bacterial burden at multiple time points after infection would substantially strengthen the conclusions and provide additional insight into how native Wolbachia improves host survival in this case.

We agree that this is an important issue and have raised it in the discussion (lines 542--545). We are currently unable to repeat the assay to study bacterial load and clearance over time post-challenge, but agree that it would be an interesting future research direction, which we also note in the study limitation section (lines 659-662).

3. The interpretation of differences between gram-positive and gram-negative bacteria should be treated with greater caution. Authors frequently attribute observed differences to bacterial gram status; however, only two bacterial isolates were examined within each category, also noted by the authors in limitations section. Species-specific characteristics unrelated to gram classification could equally explain the observed differences in mortality and antiviral effects. Therefore, my suggestion will be to adjust the conclusions by not just generalizing to gram aspect of the bacteria but also the bacterial species examined.

We have tempered the language relating to bacterial gram status throughout the text, and noted in the discussion that there is a need to assess these effects for a broader range of bacteria but also that the effects we observed in our study may not persist if other bacteria, regardless of gram status, were to be tested in the same system (lines 594-597). This issue is also mentioned in the study limitations section.

4. For DENV vector competence assays, authors note that only mosquitoes that survived the initial bacterial challenge were subsequently used for dengue assay. Their approach likely represents a non-random subset of individuals with greater physiological robustness or stronger immune function. It may consequently affect the reduced viral susceptibility independently of immune priming. The authors should explicitly acknowledge and discuss this potential source of bias.

This is an important point that we have mentioned in the discussion section (lines 564-568)

5. Did authors check what happens to Wolbachia density/titers upon bacterial and viral exposures? There are several studies that reflect negative impact on Wolbachia by certain bacterial and viral strains depending on the host, Wolbachia, and pathogen combination. This insight could provide valuable context for explaining some of their data.

Unfortunately, we were not able to check this issue at the time experiments were performed, but we have now mentioned it in the study limitations section (lines 648-650).

Minor comments:

Methods: Specify how many generations elapsed between tetracycline treatment, flora restoration, and experimental assays.

This information has been added to the methods section (lines 199-201).

Clarify the rationale for selecting a 5-day interval between bacterial challenge and DENV exposure.

This has been added to the text (lines 298-301).

Similarly, why 14dpi for checking DENV prevalence? Add appropriate citations if this timeline is established in literature.

This is a common time point for examining DENV infections in Aedes mosquitoes because it is at the upper limit of the DENV extrinsic incubation period, when the virus has likely had sufficient time to invade the mosquito salivary glands in the majority of exposed mosquitoes. We have added this rationale and supporting references to the text (lines 311-312).

Reviewer #3: In this study, the authors investigate dual bacterial infections in the major vector mosquito Aedes albopictus. Wolbachia infection was found to reduce mortality from other bacterial infection. Further, one other type of bacterial infections when together with Wolbachia could reduce dengue uptake in the mosquito, which could potentially affect transmission dynamics.

For context, I don’t specialize in the molecular mechanisms of Wolbachia, but I am familiar with it and have done some related modeling.

Comments

The impact of this study is unclear to me. How often do mosquitoes get these non-Wolbachia bacterial infections in nature (it should be at least a little because of how they were found, but see next paragraph), or what would be the prospects of providing this infection? This is not introduced. If prospects for both are low, then it does not matter much if additional infections reduce dengue load.

It’s also unclear if intrathoracic injection of bacteria would provide similar results compared to natural infection by these bacteria.

Systemic microbial infections via the cuticle and/or resulting from wounding are very common in insects. Assessment of microbial infection in insects via stabbing/pricking with a needle or via injection are common methods of generating systemic infection in the literature. Both are comparable to wounding, but differ in effect from oral microbial exposure, which is another common method through which microorganisms come to associate with insects in nature. We have added this rationale and supporting references to the text (lines 235-237).

Line 22-23: This is unclear. There is no pathogen blocking in Drosophila, but that seems to be what the sentence implies. Please reword.

This sentence refers to two different methods of bacterial challenge. We have rewritten this sentence to clarify that Wolbachia-mediated bacterial blocking has been observed after oral bacterial challenge, but not after systemic/intrathoracic bacterial challenge (Line 23).

Line 142: Is mutualism really the right word here? The Wolbachia is still a parasite. It’s just that bacteria and/or host have learned to live with each other more efficiently.

Wolbachia can exhibit aspects of both parasitism and mutualism, depending on the host-strain combination, whether or not the infection is artificially generated, and their co-association history. There are several research articles and commentaries on this topic in the literature, including:

<https://www.pnas.org/doi/abs/10.1073/pnas.0911476107>

<https://journals.plos.org/plosbiology/article?id=10.1371/journal.pbio.0050114>

<https://journals.asm.org/doi/full/10.1128/jb.00589-19>

<https://onlinelibrary.wiley.com/doi/full/10.1111/brv.12098>

To support this concept, we have adjusted the wording of this sentence, which cites two studies on this topic (Lines 141-144).

Figure 1 results: It looks like the control here is also showing a difference. The tet-treated mosquitoes seem to have slightly higher mortality than the WT (Wolbachia) controls. Thus, I am not sure if you can say that the tet mosquitoes are more affected by infection than the WT controls due to presence of Wolbachia. They are already a little weaker in general, and this might mean they get even more affected by infection. It’s also odd that the tet mosquitoes are less healthy in the first place. If the Wolbachia parasite was removed and everything else was actually the same, then they should be a little more healthy unless the Wolbachia is actually doing something important for the mosquitoes that is not related to their specific response against these laboratory infections. In this case, the mechanism against the infection is still confounded. The pairwise statistical comparison that tries to address this is insufficient because it has limited power to see effects that might be important when magnified with bacterial infection (and it looks highly significant on the actual mortality graph, which is a little odd for such a high p-value).

We agree that this is an issue that is definitely relevant to the interpretation of results from both experiments. For native Wolbachia infections in insects, it is not uncommon to find that an aposymbiotic line, living without its natural symbionts, has lower fitness than the wildtype line (i.e., doi.org/10.1038/sj.hdy.6800514). This contrasts with transinfected insect lines, where fitness is typically lower in the presence of the symbiont (oi.org/10.1371/journal.pntd.0004320). We have raised this issue in the results (paragraph starting line 438) and study limitations sections (lines 655-658), but have also re-written and expanded discussion of it in the context of these two references, which we believe now better frames the issue.

Figure 2A-B: No statistical comparisons are presented for this data in the figure itself. This might not be needed if SEM or another distribution was presented (this would be simple due to binomial data but could still show viewers why things are not statistically significant).

The values presented in these panels represent absolute DENV prevalence, with the values for each bar being generated from a random sample of 20 specimens selected from the three replicate assays. As there was a small sample size from each individual assay (N = 5-8), we chose to analyze the data collectively, rather than by replicate, which means there are not any distribution values to include in the graph.

The binomial glm we used to assess differences in prevalence for mosquito bodies (panel A) revealed that the only significant effect was for priming with either of the two gram negative bacteria, and that there were no significant Wolbachia effects (either the main or interaction terms). We are confident that this effect is clear from the panel, but have revised the text in the figure legend to clarify that there are no effects of Wolbachia on DENV prevalence. For panel B, there were no significant effects. Based on the low number of significant effects across the dataset (including saliva sample prevalence from Fig. S2), we felt pairwise comparisons were unnecessary in our analysis of this parameter.

Line 442-443: Does this statistical method take multiple testing correction into account? I think it might, but please double check. There are a lot of comparisons that were done in this data set, and only one piece of data was statistically significant. This might go away after multiple testing correction.

As mentioned in our response above, we felt that it was unnecessary to conduct pairwise comparisons for these data due to the low number of significant effects. You are correct in that only one term in this glm has a significant P value, which was 0.00757. In our experience, it is not necessary to run multiple test corrections on the output of a single general linear model, however, if we were to run a 5% false discovery rate on those data, the adjusted P value for that term would still be significant. We are confident in the analysis of these data as they stand in the text.

Line 533: I thought there were no statistically significant results from dengue prevalence?

There were no significant effects due to Wolbachia, but DENV prevalence was significantly reduced after an initial challenge with one of the two gram negative bacterial isolates. We are comfortable that the text in this part of the manuscript (now lines 552-556) accurately defines our findings.

Line 561-562: I thought only Brevundimonas sp. bacteria gave a statistically significant result (and only when infected with Wolbachia)?

In our model for DENV load in mosquito bodies, significant reductions in DENV load were observed for both gram positive bacteria and gram negative bacteria, as well as the gram negative by Wolbachia infection status interaction terms. We have re-written the relevant part of the results section to better clarify this (Lines 504-509).
