# Supplementary material for: Bacterial immune priming and Wolbachia-mediated dengue blocking in Aedes albopictus mosquitoes
Source: PLoS Negl Trop Dis. 2026 Oct 16;20(10):e0014807. doi: 10.1371/journal.pntd.0014807 (PMC13712725; doi:10.1371/journal.pntd.0014807)
Supplement: Peer review history

# Peer review: Decision Letter 0

**DOI:** 10.1371/journal.pntd.0014807.r001

**Authors:** Amy C. Morrison, James Lee Crainey

18 Aug 2026

Response to Reviewers Revised Manuscript with Track Changes Manuscript

Shaden Kamhawi

co-Editor-in-Chief

PLOS Neglected Tropical Diseases

orcid.org/0000-0003-4304-636XX

Paul Brindley

co-Editor-in-Chief

PLOS Neglected Tropical Diseases

orcid.org/0000-0003-1765-0002

**Additional Editor Comments (if provided):** **Journal Requirements:**

1) We ask that a manuscript source file is provided at Revision. Please upload your manuscript file as a .doc, .docx, .rtf or .tex. If you are providing a .tex file, please upload it under the item type u2018LaTeX Source Fileu2019 and leave your .pdf version as the item type u2018Manuscriptu2019.

2) We have noticed that you have uploaded Supporting Information files, but you have not included a list of legends. Please add a full list of legends for your Supporting Information files after the references list.

3) Please amend your detailed Financial Disclosure statement. This is published with the article. It must therefore be completed in full sentences and contain the exact wording you wish to be published.

i) State what role the funders took in the study. If the funders had no role in your study, please state: "The funders had no role in study design, data collection and analysis, decision to publish, or preparation of the manuscript."

ii) If any authors received a salary from any of your funders, please state which authors and which funders..

If you did not receive any funding for this study, please simply state: u201cThe authors received no specific funding for this work.u201d

4) We have amended your Competing Interest statement to comply with journal style. We kindly ask that you double check the statement and let us know if anything is incorrect.

**Reviewers' comments:**

**Key Review Criteria Required for Acceptance?**

As you describe the new analyses required for acceptance, please consider the following:

**Methods**

-Are the objectives of the study clearly articulated with a clear testable hypothesis stated?

-Is the study design appropriate to address the stated objectives?

-Is the population clearly described and appropriate for the hypothesis being tested?

-Is the sample size sufficient to ensure adequate power to address the hypothesis being tested?

-Were correct statistical analysis used to support conclusions?

-Are there concerns about ethical or regulatory requirements being met?

Reviewer #1: The objective is clear, with the hypothesis that infection with a sublethal dose of bacteria prior to infection would result in greater protection against dengue virus in mosquitoes, and that there may be an interaction with Wolbachia. The design is appropriate, with clear descriptions, adequate sample sizes and statistics.

Reviewer #2: (No Response)

Reviewer #3: (No Response)

**********

**Results**

-Does the analysis presented match the analysis plan?

-Are the results clearly and completely presented?

-Are the figures (Tables, Images) of sufficient quality for clarity?

Reviewer #1: The analysis and plan do match, which both focus on determining if infection with bacteria results in an impact on subsequent dengue infection. The results and figures are mostly clearly presented, with only minor suggestions for clarity in the comments below.

Reviewer #2: (No Response)

Reviewer #3: (No Response)

**********

**Conclusions**

-Are the conclusions supported by the data presented?

-Are the limitations of analysis clearly described?

-Do the authors discuss how these data can be helpful to advance our understanding of the topic under study?

-Is public health relevance addressed?

Reviewer #1: The conclusions are largely supported by the data, and the authors do a thorough job explaining the relevant limitations. They make a clear case in the discussion for how this information helps advance our understanding of the interaction between immunity and Wolbachia blocking of mosquito-borne pathogens, which is of great relevance to human health. I do have some comments on the wording specifically and what it implies about mechanism, addressed below.

Reviewer #2: (No Response)

Reviewer #3: (No Response)

**********

**Editorial and Data Presentation Modifications?**

Use this section for editorial suggestions as well as relatively minor modifications of existing data that would enhance clarity. If the only modifications needed are minor and/or editorial, you may wish to recommend “Minor Revision” or “Accept”.

Reviewer #1: • Figures 1&2: include gram status in figure for clarity- the text refers to effects of gram positive vs negative microbes, but the reader has to reference the text and go back and forth to the figure. It would be better to have the gram status labelled on the figure to compare to the conclusions in the text.

• Fig 2C/D: The statistical bars (median, etc.) are difficult to see. Please modify so that they lie on top of the dots. Same for the S2B plot.

Reviewer #2: (No Response)

Reviewer #3: (No Response)

**********

**Summary and General Comments**

Use this section to provide overall comments, discuss strengths/weaknesses of the study, novelty, significance, general execution and scholarship. You may also include additional comments for the author, including concerns about dual publication, research ethics, or publication ethics. If requesting major revision, please articulate the new experiments that are needed.

Reviewer #1: This manuscript by Alomar et al. assesses whether bacterial immune priming in Aedes mosquitoes results in any measurable impact on Wolbachia’s interactions with dengue virus in the host. Here, the authors use several bacterial pathogens isolated from mosquitoes and demonstrate that low-dose infections can measurably increase protection against subsequent dengue virus when considering viral load. This is moderately enhanced by the Wolbachia symbiont. The strength is in the experimental design and analysis, which is rigorous. The weaknesses, acknowledged by the authors, are that the study is limited in scope and that the phenotype/effects measured are fairly moderate in strength. However, it is beneficial, as immune priming is poorly understood in the context of Wolbachia. The paper is straightforward, with good experimental design, and demonstrates results from a couple of key experiments.

A few general questions/comments for the authors:

• Immune priming:

o Is it known if this mosquito typically clears bacterial infection within the timeline in this study?

o L501: careful with wording in cases like this- “resistance” might be misread as immune resistance which was not tested here

o It may help to discuss hypotheses other than immune priming here since the priming has not been more directly tested. For example, it may have to do with altered physiology or microbiome or nutrient status post-bacterial infection that then impacts dengue virus. Sometimes the wording in the manuscript makes it seem like immune priming is the definite mechanism here, which it is likely to be, but not yet proven. Since it is in the title/abstract etc., I would suggest some rewording and further discussion (maybe “consistent with immune priming” or “immune priming-like”, etc.). I thought the wording of “bacterial priming” that was often used was a good alternative.

Reviewer #2: The study by Alomar et al. asks an interesting question whether native Wolbachia provides anti-bacterial pathogen protection and whether bacterial immune priming interacts with native Wolbachia-conferred pathogen blocking in Ae. Albopictus. The experiments are generally well executed. Authors did a good job acknowledging the limitations of their assays. However, the central claim that revolves around immune priming is not fully supported by the presented data. See my comments below:

Major comments:

1. My biggest concern is that "Immune priming" is assumed rather than demonstrated in the study. Immune priming is more than exposing insects to bacteria before another infection. Authors exposed mosquitoes to a sublethal bacterial infection followed by DENV challenge five days later, but they do not establish whether the initial bacterial infections had been cleared before viral exposure. Consequently, the observed reduction in viral prevalence or load could equally reflect persistent bacterial infection, chronic immune activation, altered metabolism, or physiological stress rather than an induced memory-like immune state. Throughout the manuscript, the authors interpret these effects as immune priming without directly demonstrating that a primed immune state exists. They could have measured bacterial loads immediately before and 5 days after injection. If bacteria persist 5 days after injection, the subsequent viral challenge would represent a simultaneous bacterial-viral co-infection rather than an immune priming experiment. Making this distinction is critical for interpretation of the results.

2. The authors show that Wolbachia-infected mosquitoes survive longer after bacterial challenge, but it remains unclear whether this reflects enhanced bacterial clearance (resistance) or increased tolerance to bacterial infection. Since theirs is the first study measuring native Wolbachia effect in Ae. Albopictus, measuring bacterial burden at multiple time points after infection would substantially strengthen the conclusions and provide additional insight into how native Wolbachia improves host survival in this case.

3. The interpretation of differences between gram-positive and gram-negative bacteria should be treated with greater caution. Authors frequently attribute observed differences to bacterial gram status; however, only two bacterial isolates were examined within each category, also noted by the authors in limitations section. Species-specific characteristics unrelated to gram classification could equally explain the observed differences in mortality and antiviral effects. Therefore, my suggestion will be to adjust the conclusions by not just generalizing to gram aspect of the bacteria but also the bacterial species examined.

4. For DENV vector competence assays, authors note that only mosquitoes that survived the initial bacterial challenge were subsequently used for dengue assay. Their approach likely represents a non-random subset of individuals with greater physiological robustness or stronger immune function. It may consequently affect the reduced viral susceptibility independently of immune priming. The authors should explicitly acknowledge and discuss this potential source of bias.

5. Did authors check what happens to Wolbachia density/titers upon bacterial and viral exposures? There are several studies that reflect negative impact on Wolbachia by certain bacterial and viral strains depending on the host, Wolbachia, and pathogen combination. This insight could provide valuable context for explaining some of their data.

Minor comments:

Methods: Specify how many generations elapsed between tetracycline treatment, flora restoration, and experimental assays.

Clarify the rationale for selecting a 5-day interval between bacterial challenge and DENV exposure.

Similarly, why 14dpi for checking DENV prevalence? Add appropriate citations if this timeline is established in literature.

Reviewer #3: In this study, the authors investigate dual bacterial infections in the major vector mosquito Aedes albopictus. Wolbachia infection was found to reduce mortality from other bacterial infection. Further, one other type of bacterial infections when together with Wolbachia could reduce dengue uptake in the mosquito, which could potentially affect transmission dynamics.

For context, I don’t specialize in the molecular mechanisms of Wolbachia, but I am familiar with it and have done some related modeling.

Comments

The impact of this study is unclear to me. How often do mosquitoes get these non-Wolbachia bacterial infections in nature (it should be at least a little because of how they were found, but see next paragraph), or what would be the prospects of providing this infection? This is not introduced. If prospects for both are low, then it does not matter much if additional infections reduce dengue load.

It’s also unclear if intrathoracic injection of bacteria would provide similar results compared to natural infection by these bacteria.

Line 22-23: This is unclear. There is no pathogen blocking in Drosophila, but that seems to be what the sentence implies. Please reword.

Line 142: Is mutualism really the right word here? The Wolbachia is still a parasite. It’s just that bacteria and/or host have learned to live with each other more efficiently.

Figure 1 results: It looks like the control here is also showing a difference. The tet-treated mosquitoes seem to have slightly higher mortality than the WT (Wolbachia) controls. Thus, I am not sure if you can say that the tet mosquitoes are more affected by infection than the WT controls due to presence of Wolbachia. They are already a little weaker in general, and this might mean they get even more affected by infection. It’s also odd that the tet mosquitoes are less healthy in the first place. If the Wolbachia parasite was removed and everything else was actually the same, then they should be a little more healthy unless the Wolbachia is actually doing something important for the mosquitoes that is not related to their specific response against these laboratory infections. In this case, the mechanism against the infection is still confounded. The pairwise statistical comparison that tries to address this is insufficient because it has limited power to see effects that might be important when magnified with bacterial infection (and it looks highly significant on the actual mortality graph, which is a little odd for such a high p-value).

Figure 2A-B: No statistical comparisons are presented for this data in the figure itself. This might not be needed if SEM or another distribution was presented (this would be simple due to binomial data but could still show viewers why things are not statistically significant).

Line 442-443: Does this statistical method take multiple testing correction into account? I think it might, but please double check. There are a lot of comparisons that were done in this data set, and only one piece of data was statistically significant. This might go away after multiple testing correction.

Line 533: I thought there were no statistically significant results from dengue prevalence?

Line 561-562: I thought only Brevundimonas sp. bacteria gave a statistically significant result (and only when infected with Wolbachia)?

**********

PLOS authors have the option to publish the peer review history of their article (what does this mean?). If published, this will include your full peer review and any attached files.

If you choose “no”, your identity will remain anonymous but your review may still be made public.

**Do you want your identity to be public for this peer review?**  For information about this choice, including consent withdrawal, please see our Privacy Policy.

Reviewer #1: No

Reviewer #2: No

Reviewer #3: No

**Figure resubmission:**

**Reproducibility:**

To enhance the reproducibility of your results, we recommend that authors of applicable studies deposit laboratory protocols in protocols.io, where a protocol can be assigned its own identifier (DOI) such that it can be cited independently in the future. Additionally, PLOS ONE offers an option to publish peer-reviewed clinical study protocols. Read more information on sharing protocols at https://plos.org/protocols?utm_medium=editorial-email&utm_source=authorletters&utm_campaign=protocols

# Author comment: Author response to Decision Letter 1

**DOI:** 10.1371/journal.pntd.0014807.r002

17 Sep 2026

Attachment Submitted filename: Response to reviewers.docx

# Peer review: Decision Letter 1

**DOI:** 10.1371/journal.pntd.0014807.r003

**Authors:** Amy C. Morrison, James Lee Crainey

6 Oct 2026

Dear Dr Caragata,

We are pleased to inform you that your manuscript 'Bacterial immune priming and Wolbachia-mediated dengue blocking in Aedes albopictus mosquitoes' has been provisionally accepted for publication in PLOS Neglected Tropical Diseases.

Before your manuscript can be formally accepted you will need to complete some formatting changes, which you will receive in a follow up email. A member of our team will be in touch with a set of requests.

Please note that your manuscript will not be scheduled for publication until you have made the required changes, so a swift response is appreciated.

IMPORTANT: The editorial review process is now complete. PLOS will only permit corrections to spelling, formatting or significant scientific errors from this point onwards. Requests for major changes, or any which affect the scientific understanding of your work, will cause delays to the publication date of your manuscript.

Should you, your institution's press office or the journal office choose to press release your paper, you will automatically be opted out of early publication. We ask that you notify us now if you or your institution is planning to press release the article. All press must be co-ordinated with PLOS.

Thank you again for supporting Open Access publishing; we are looking forward to publishing your work in PLOS Neglected Tropical Diseases.

Best regards,

James Lee Crainey, Ph.D.

Academic Editor

PLOS Neglected Tropical Diseases

Amy Morrison

Section Editor

PLOS Neglected Tropical Diseases

Shaden Kamhawi

co-Editor-in-Chief

PLOS Neglected Tropical Diseases

orcid.org/0000-0003-4304-636XX

Paul Brindley

co-Editor-in-Chief

PLOS Neglected Tropical Diseases

orcid.org/0000-0003-1765-0002

***********************************************************

As of today, we have all three reviewers´ comments on your revised manuscript; I am, therefore, now, like two of your three referees, satisfied that your paper is suitable for publication in PLOS NTDs. While I do agree with referee #3 that some of their comments could have been more fully addressed, I don´t think any of the outstanding issues they have highlighted can justify delaying the publication of this important work any further. I, therefore, wish only to congratulate the authors on their fine work and thank them for their patience during the extraordinarily long reviewer recruitment period.

Reviewer's Responses to Questions

**Key Review Criteria Required for Acceptance?**

As you describe the new analyses required for acceptance, please consider the following:

**Methods**

-Are the objectives of the study clearly articulated with a clear testable hypothesis stated?

-Is the study design appropriate to address the stated objectives?

-Is the population clearly described and appropriate for the hypothesis being tested?

-Is the sample size sufficient to ensure adequate power to address the hypothesis being tested?

-Were correct statistical analysis used to support conclusions?

-Are there concerns about ethical or regulatory requirements being met?

Reviewer #1: The methods and study design are clear, and the authors thoroughly answered all reviewer questions related to the methods.

Reviewer #2: (No Response)

Reviewer #3: (No Response)

**********

**Results**

-Does the analysis presented match the analysis plan?

-Are the results clearly and completely presented?

-Are the figures (Tables, Images) of sufficient quality for clarity?

Reviewer #1: Results are clear, analysis is aligned.

Reviewer #2: (No Response)

Reviewer #3: (No Response)

**********

**Conclusions**

-Are the conclusions supported by the data presented?

-Are the limitations of analysis clearly described?

-Do the authors discuss how these data can be helpful to advance our understanding of the topic under study?

-Is public health relevance addressed?

Reviewer #1: The revised conclusions are now supported by the data presented and the authors cover the major caveats. The importance of the study is also clear relating to Wolbachia-based vector control.

Reviewer #2: (No Response)

Reviewer #3: (No Response)

**********

**Editorial and Data Presentation Modifications?**

Use this section for editorial suggestions as well as relatively minor modifications of existing data that would enhance clarity. If the only modifications needed are minor and/or editorial, you may wish to recommend “Minor Revision” or “Accept”.

Reviewer #1: Two very minor comments:

1) The figures are low resolution and may need to be updated.

2) The title still has "immune" in it. I'd suggest changing to just "Bacterial priming..." based on reviewer comments

Reviewer #2: (No Response)

Reviewer #3: (No Response)

**********

**Summary and General Comments**

Use this section to provide overall comments, discuss strengths/weaknesses of the study, novelty, significance, general execution and scholarship. You may also include additional comments for the author, including concerns about dual publication, research ethics, or publication ethics. If requesting major revision, please articulate the new experiments that are needed.

Reviewer #1: The authors thoroughly responded to all comments and incorporated reasonable changes to address them in the revised manuscript. It is now in very good overall shape.

Reviewer #2: Although authors haven't done extra analysis to address a couple of my major comments, they have acknowledged and modified the text to reflect those limitations.

Reviewer #3: The authors have done a good job providing answers to previous points, and my apologies for missing a couple key words in my previous read that could have preventing a couple comments. I do have some remaining questions, though.

“>” Indicates previous review comments and “>>” indicates responses.

>The impact of this study is unclear to me. How often do mosquitoes get these non-

Wolbachia bacterial infections in nature (it should be at least a little because of how they

were found, but see next paragraph), or what would be the prospects of providing this

infection? This is not introduced. If prospects for both are low, then it does not matter

much if additional infections reduce dengue load.

It’s also unclear if intrathoracic injection of bacteria would provide similar results

compared to natural infection by these bacteria.

>>Systemic microbial infections via the cuticle and/or resulting from wounding are very

common in insects. Assessment of microbial infection in insects via stabbing/pricking

with a needle or via injection are common methods of generating systemic infection in

the literature. Both are comparable to wounding, but differ in effect from oral microbial

exposure, which is another common method through which microorganisms come to

associate with insects in nature. We have added this rationale and supporting

references to the text (lines 235-237).

This response only addresses a small part of my concern. It’s fine to assume that wounding and infection happens in nature. The is very sensible. The question is: does this occur to an extent that it may become important for disease dynamics? Further, it’s fine to use standard laboratory models for infection in many cases, but here you need to mimic a very specific process in the wild involving pathogen interaction.

>Figure 1 results: It looks like the control here is also showing a difference. The tet-treated

mosquitoes seem to have slightly higher mortality than the WT (Wolbachia)

controls. Thus, I am not sure if you can say that the tet mosquitoes are more affected by

infection than the WT controls due to presence of Wolbachia. They are already a little

weaker in general, and this might mean they get even more affected by infection. It’s

also odd that the tet mosquitoes are less healthy in the first place. If the Wolbachia

parasite was removed and everything else was actually the same, then they should be a

little more healthy unless the Wolbachia is actually doing something important for the

mosquitoes that is not related to their specific response against these laboratory

infections. In this case, the mechanism against the infection is still confounded. The

pairwise statistical comparison that tries to address this is insufficient because it has

limited power to see effects that might be important when magnified with bacterial

infection (and it looks highly significant on the actual mortality graph, which is a little odd

for such a high p-value).

>>We agree that this is an issue that is definitely relevant to the interpretation of results

from both experiments. For native Wolbachia infections in insects, it is not uncommon to

find that an aposymbiotic line, living without its natural symbionts, has lower fitness than

the wildtype line (i.e., doi.org/10.1038/sj.hdy.6800514). This contrasts with transinfected

insect lines, where fitness is typically lower in the presence of the symbiont

(oi.org/10.1371/journal.pntd.0004320). We have raised this issue in the results

(paragraph starting line 438) and study limitations sections (lines 655-658), but have

also re-written and expanded discussion of it in the context of these two references,

which we believe now better frames the issue.

This can sort of explain the difference between controls, but it just reframes the issue. If the Wolbachia is a bit mutualistic (a reasonable proportions based on other responses), then one can argue that Wolbachia is making the mosquitoes generally healthier, which affects their ability to respond to infection, rather than a specific infection-response specific effect. This is probably worth discussing.

Further, I noticed that the CPH seems to produce a reasonable-looking statistical significance for the effect of Wolbachia in general (line 386), just barely significant, in line with the error bars in Figure 1H (all overlapping with mock, but together showing a pattern). However, later in the paragraph, extremely small p values are reported for individual infections. How could this be? It seems to be about the same thing: the effect of absence of Wolbachia, just for individual infections (less power) than everything together.

**********

PLOS authors have the option to publish the peer review history of their article (what does this mean?). If published, this will include your full peer review and any attached files.

If you choose “no”, your identity will remain anonymous but your review may still be made public.

**Do you want your identity to be public for this peer review?**  For information about this choice, including consent withdrawal, please see our Privacy Policy.

Reviewer #1: No

Reviewer #2: No

Reviewer #3: No

# Editor report: Acceptance letter

**DOI:** 10.1371/journal.pntd.0014807.r004

**Authors:** Amy C. Morrison, James Lee Crainey

Dear Dr Caragata,

We are delighted to inform you that your manuscript, "Bacterial immune priming and Wolbachia-mediated dengue blocking in Aedes albopictus mosquitoes," has been formally accepted for publication in PLOS Neglected Tropical Diseases.

We have now passed your article onto the PLOS Production Department who will complete the rest of the publication process. All authors will receive a confirmation email upon publication.

The corresponding author will soon be receiving a typeset proof for review, to ensure errors have not been introduced during production. Please review the PDF proof of your manuscript carefully, as this is the last chance to correct any scientific or type-setting errors. Please note that major changes, or those which affect the scientific understanding of the work, will likely cause delays to the publication date of your manuscript. Note: Proofs for Front Matter articles (Editorial, Viewpoint, Symposium, Review, etc...) are generated on a different schedule and may not be made available as quickly.

Soon after your final files are uploaded, the early version of your manuscript will be published online unless you opted out of this process. The date of the early version will be your article's publication date. The final article will be published to the same URL, and all versions of the paper will be accessible to readers.

For Research Articles, you will receive an invoice from PLOS for your publication fee after your manuscript has reached the completed accept phase. If you receive an email requesting payment before acceptance or for any other service, this may be a phishing scheme. Learn how to identify phishing emails and protect your accounts at https://explore.plos.org/phishing.

Thank you again for supporting open-access publishing; we are looking forward to publishing your work in PLOS Neglected Tropical Diseases.

Best regards,

Shaden Kamhawi

co-Editor-in-Chief

PLOS Neglected Tropical Diseases

Paul Brindley

co-Editor-in-Chief

PLOS Neglected Tropical Diseases
